# Need for (expected) speed: Exploring the indirect influence of trial type consistency on representational momentum

**DOI:** 10.3758/s13414-023-02796-0

**Published:** 2023-10-11

**Authors:** Simon Merz, Charles Spence, Christian Frings

**Affiliations:** 1 Department of Psychology, Cognitive Psychology, University of Trier, Universitätsring 15, 54286 Trier, Germany; 2 Department of Experimental Psychology, University of Oxford, Oxford, UK

**Keywords:** Representational momentum, Trial type consistency, Vision, Motion perception

## Abstract

The biases affecting people’s perception of dynamic stimuli are typically robust and strong for specific stimulus configurations. For example, representational momentum describes a systematic perceptual bias in the direction of motion for the final location of a moving stimulus. Under clearly defined stimulus configurations (e.g., specific stimulus identity, size, speed), for example, the frequently used “implied motion” trial sequence, for which a target is subsequently presented in a consistent direction and with a consistent speed, a displacement in motion direction is evidenced. The present study explores the potential influence of expectations regarding directional as well as speed consistencies on representational momentum, elicited by including other, inconsistently moving trial types within the same experimental block. A systematic representational momentum effect was observed when only consistent motion trials were presented. In contrast, when inconsistent target motion trials were mixed within the same block of experimental trials, the representational momentum effect decreased, or was even eliminated (Experiments 1 & 2). Detailed analysis indicated that this reflects a global (proportion of consistent and inconsistent motion trials within a particular experimental block), not local (preceding trial influencing actual trial) effect. Yet, additional follow-up studies (Experiments 3 & 4) support the idea that these changes in perceived location are strongly influenced by the overall stimulus speed statistics in the different experimental blocks. These results are discussed and interpreted in light of recent theoretical developments in the literature on motion perception that highlight the importance of expectations about stimulus speed for motion perception.

## Introduction

In our everyday lives, we are surrounded by stimuli that change and move. As such, it is perhaps unsurprising that the perception of dynamic information has been investigated by researchers for more than a century (Fröhlich, 1923; Wertheimer, 1912). Yet, to date, the mechanisms underlying the perception of dynamic information are still unclear, and a number of different accounts have been put forward to explain several of the fundamental biases that have been shown to affect the perception of moving stimuli (Angelaki et al., 2004; Hubbard, 2010; Jancke & Erlhagen, 2010; Merz et al., 2022; Pei & Bensmaia, 2014; Weiss & Adelson, 1998). One of these biases, the so-called representational momentum phenomenon, is a typical forward shift, for which the final location of a stimulus is misperceived in the direction of motion (Freyd & Finke, 1984). To date, this phenomenon has been extensively investigated and several moderating influences have been observed (Hubbard, 2014, 2018), and different theoretical frameworks have been proposed (for an extensive review, see Hubbard, 2010; for our new approach, see Merz et al., 2022). The present study adds a new influence on the representational momentum phenomenon, namely expectations regarding the stimulus characteristics within a given experimental block, by using one of the main motion patterns known in the literature, the implied motion sequence.

**Fig. 1:**
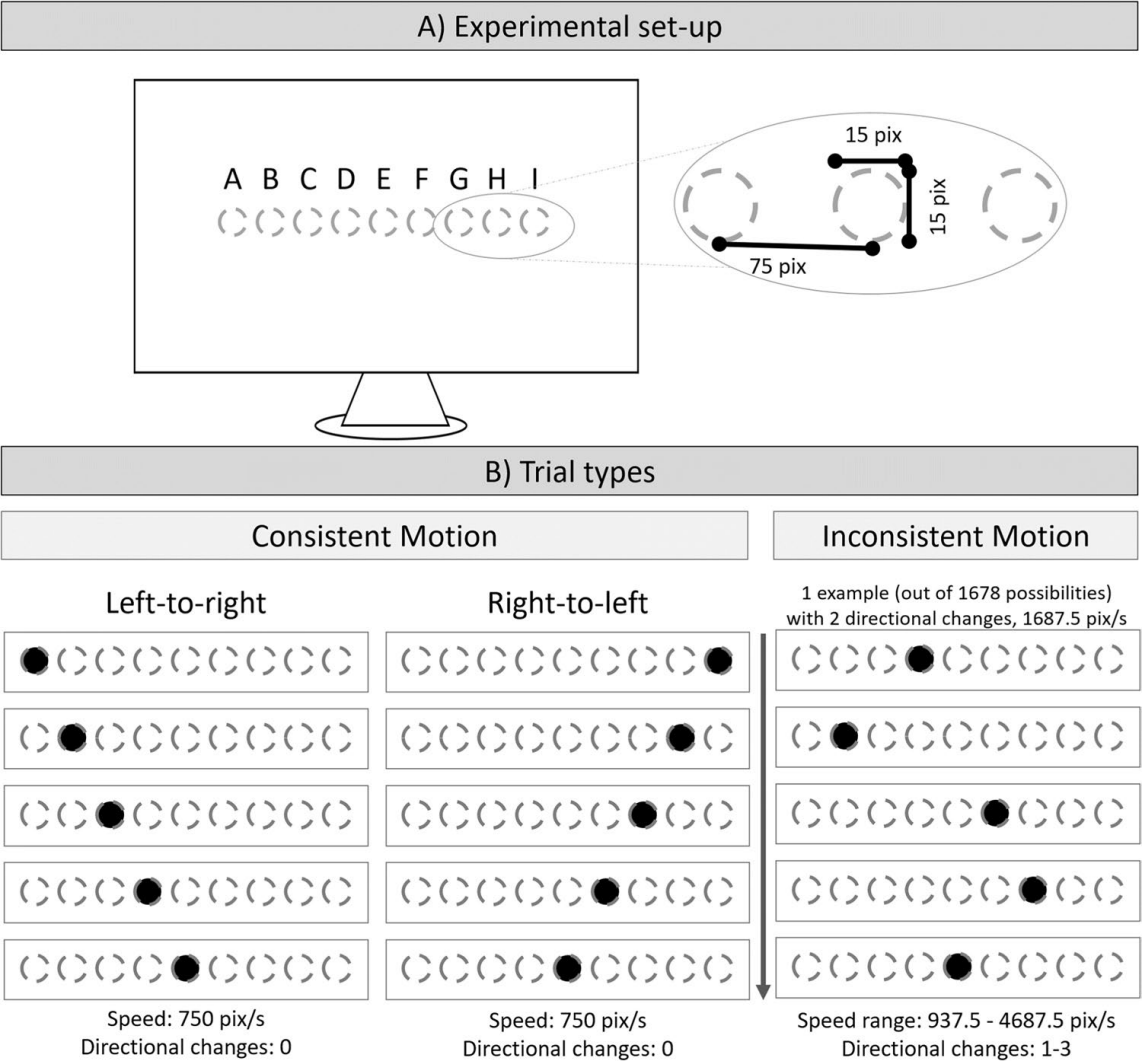
Graphical depiction of the experimental set-up **A**) as well as the trial types **B**), consistent motion and inconsistent motion trials. Importantly, only the filled circles are visible to the participants, the dotted circles are presented to visualize all possible target positions, yet these positions were not specified to the participants. For consistent trials, two different directions were possible (left-to-right, as well as right-to-left). For the inconsistent trials, only one possible trial sequence is depicted, out of the 1678 possible trial sequences. See the main text for more information

The implied motion stimuli that have typically been used in the representational momentum literature involve a target stimulus (e.g., a circle) which is presented several times (often three or five times, e.g., Freyd & Finke, 1984; Hubbard, 1993; Hubbard & Ruppel, 2014) at successive, adjacent locations (for a visualization, see Fig. 1). The spacing between and timing of successive target presentations is kept constant to elicit a consistent movement in one direction and with one speed, and subsequently, we will refer to this sequence as the consistent motion sequence. The consistency of this motion sequence is an important precondition for representational momentum to occur. Spatial changes in inconsistent directions,^1^ as well as inconsistent spacing between successive presentations, have long been known not to give rise to the representational momentum phenomenon (Freyd & Finke, 1984).^2^ Therefore, such inconsistent motion trials are often used as a control measurement, especially in tactile studies (Merz et al., 2019a, b, 2022, for similar approaches in audition, see Getzmann & Lewald, 2007, 2009). Interestingly, most often these trials were included within the same experimental block in which the consistent motion trials were presented. Although this might seem like a small detail, the evidence suggests that mixing the two trial types leads to systematic changes in representational momentum for consistent motion trials in touch (Merz et al., 2023), which have also been observed in vision (the data from two pilot studies are reported in the online supplement uploaded to the projects OSF page). We deem this as an indirect effect of trial type consistency, as the analyzed trials were always the consistent motion pattern, therefore the effect is indirect in that sense that the mere inclusion of inconsistent motion trial within the same experimental block results in perceptual changes for the consistent motion trials. Yet, the nature of this new indirect effect of trial type consistency has not yet been investigated, which is the goal of the present study.

In an experimental block with both consistent and inconsistent motion trial types mixed together, it is likely that expectations regarding actual to-be-encountered target behavior will be different compared with an experimental block that contain only consistent motion trials. To elaborate, consistent motion trials typically involve the successive presentation of the same target stimulus at adjacent, evenly-spaced locations, indicating a consistent speed in a consistent direction. In contrast, for the inconsistent trials, the spatial locations don’t need to be adjacent or in one consistent direction. That is, larger spatial jumps between successive presentations are likely, resulting in overall faster stimulus displays. Directional changes within the target sequence are also possible (e.g., Freyd & Finke, 1984; Merz et al., 2019a, b, 2022). We propose that these inconsistencies lead to participants forming different expectations regarding typical target behavior in mixed experimental blocks, subsequently influencing perceived location of the consistent motion target stimulus. Therefore, the present study has two goals:

First, the effect of trial type manipulation needs to be explored in more detail. It needs to be established how exactly the inclusion of the inconsistent motion trials change perceived location. These changes might occur on a local level (based on the inconsistency the system encountered in the previous trial) or on a global level (based on the overall consistency within the experimental block), or both. One possibility is that if the perceptual system just encountered an inconsistent target motion pattern on the preceding trial, it adapts its expectation for the upcoming trial. This would then indicate an effect of local trial type consistency. Alternatively, the perceptual system might adjust expectations based on the overall statistics/likelihood of events in the experimental block, making it an effect of global trial type consistency.

Second, the indirect effect of trial type consistency needs to be explained and it needs to be understood why the introduction of inconsistent trials types results in a perceptual change of consistent motion trials. Inconsistent motion patterns are different compared with the consistent motion stimuli in two regards: motion direction as well as implied speed. Therefore, the perceptual system might be tuned to the speed of the inconsistent trials to adjust the expectation about the to-be-encountered target stimulus speed within one experimental block. Alternatively, the perceptual system might be tuned to the directional properties of the target stimulus, and expectations are formed about the typical directional changes within one experimental block.

## Influence of expectations on representational momentum

For instance, considering the anticipated future behavior of the target (after stimulus offset), forward displacement is only observed when the expected target motion was projected to continue in the same direction, rather than when the target was expected to reverse its course, indicating that forward displacement is only observed in the expected direction of target motion (e.g., Hubbard & Bharucha, 1988; similarly, see Johnston & Jones, 2006; Verfaillie & d’Ydewalle, 1991). Furthermore, expectations concerning a moving stimulus either bouncing off a wall or crashing though it results in differences of forward shifts (Hubbard, 1994). Similarly, target identity has also been shown to influence representational momentum (Reed & Vinson, 1996; Vinson & Reed, 2002), for example, with rockets expected to move very rapidly, but cathedrals not expected to move at all, visual depictions of both resulted in the classical representational momentum phenomenon (for the rocket) or not (for the cathedral). While these expectation-based effects indicate effects about future (after stimulus offset, e.g., expectation of continuous/change of direction after stimulus offset) or prototypical (typical motion pattern for prototypical objects, e.g., fast velocities for rockets) motion, the present study is concerned with expectations regarding actual, to-be-encountered target behavior. And yet, the expectation-based results from prototypical motion (Reed & Vinson, 1996; Vinson & Reed, 2002) in particular might be taken to indicate that speed expectations regarding the to-be-encountered target stimulus are used by the perceptual system. That is, while encountering the prototypical depiction of an object (e.g., a rocket), an observer might build up expectations regarding the object’s typical speed (e.g., fast). As such, these results might be seen as providing support for the speed expectation hypothesis. Furthermore, recent theoretical developments are in line with speed expectations as a moderating influence for motion perception—that is, a speed prior account of motion perception was recently introduced by us in the literature (Merz et al., 2022).

The speed prior account explains the localization of dynamic stimuli by combining a priori speed expectation about actual stimulus speed with the actual, but noisy perceptual input, thus giving rise to the final percept (for related arguments, see Goldreich, 2007; Stocker & Simoncelli, 2006; Weiss & Adelson, 1998). Following this perspective, a potential influence of mixing inconsistent trials within the same experimental block as consistent motion trials can be accounted for, and is predicted by, this account. That is, with changing (speed) expectations in different experimental blocks due to the inclusion of inconsistent motion trials (indicating different speeds), the observed perceptual shifts should also change. Therefore, the speed prior account or any other, speed-expectation-based theory would predict a moderating influence of speed on motion perception.

### The present study

The goal of the present study was therefore to explore the nature of the indirect influence of trial type consistency, and subsequent changes in expectations regarding actual, to-be-encountered target motion, on representational momentum. As the consistent motion sequence constitutes the typical motion sequence to investigate representational momentum, we will only analyze and compare these perceptually identical trials across the different experimental blocks. Two prestudies were conducted with linear, consistent motion sequences of three (e.g., comparable with Freyd & Finke, 1984; Munger et al., 2005) and five (e.g., comparable to Hubbard, 1990) target presentations. The results indicated the existence of an indirect effect of trial type consistency on representational momentum, as consistent motion trials were perceived differently in a context when inconsistent motion trials were mixed compared with the isolated presentation of only consistent motion trials. As these prestudies were designed to evidence the overall effect, yet not to analyze the nature of this effect, we report their results in the online supplement uploaded to the projects OSF-page to increase focus on the underlying mechanisms in the present manuscript. For all four experiments (as well as the two pilot studies), a baseline experimental block was conducted, for which only consistent motion trials (100% of consistent motion trials) were presented. Here, as so often reported in the literature, we always expect representational momentum (and, to foreshadow the results, always indicated the existence of representational momentum). Yet, the experiments differed in the compilation of the mixed trial block(s), in which consistent motion and inconsistent motion trials were presented together, in order to understand the indirect influence of trial type consistency. All experiments were conducted online.^3^

Experiments 1 and 2 were designed to explore the level at which the effect of trial type consistency occurs. Experiment 1 was designed to analyze whether local trial type consistency (experimental features of the previous trial, N-1) influence representational momentum. Hereby, the mixed condition consisted of 50% consistent motion, and 50% inconsistent motion trials. Yet, the order of the two trial types was only quasirandom. That is, they were designed in such a way that for consistent motion trials, in 50% of trials it was preceded by a consistent motion trial, whereas in the other half of trials, it was preceded by inconsistent motion trials. This manipulation allows for the analysis of a potential influence of local (trial *N* − 1) trial type consistency. Experiment 2 was designed to analyze the potential influence of global trial type consistency (overall proportion of the two trial types). Therefore, two mixed conditions were designed with either 80% consistent motion trials (and 20% inconsistent motion trials) or 20% consistent motion trials (and 80% inconsistent motion trials). To foreshadow the results, the data indicate a strong influence of global but no local influence of trial type consistency.

Experiment 3 was subsequently conducted to start analyzing the explanation of this indirect effect of trial type consistency. Two mixed conditions with an identical proportion of inconsistent and consistent motion trials (each with 50% consistent motion, and 50% inconsistent motion trials) were designed. Yet, the two conditions differed in their overall amount of inconsistencies regarding induced speed/direction changes by the inconsistent trials. That is, in one experimental block (high inconsistencies regarding speed and directional changes), the inconsistent trials induced more directional changes as well as faster speeds compared with a second experimental block (low inconsistencies regarding speed and directional changes). As systematic differences were observed, this is in favor of theories arguing for changes of expectations regarding motion direction or target speed to underlie the perception of dynamic objects and does not support arguments that formulate an attenuated reliance on the mechanism resulting in the representational momentum phenomena with decreasing proportions of consistent motion trials. Experiment 4 was conducted to further differentiate between the two possible expectations: expectations regarding stimulus speed or stimulus direction. Across two mixed conditions, the inconsistent trials indicated very different speed characteristics (high speed inconsistency vs. low speed inconsistency), yet the number of directional changes was kept identical for both mixed conditions. The data suggest the existence of a difference between the two experimental conditions, indicating that the different overall speeds induced by the inconsistent motion trials are the central factor underlying the observed differences in perceptual shifts.

## Experiment 1

Experiment 1 was designed to explore the indirect effect of trial type consistency in more detail, and to indicate if the effect of trial type manipulations was based on local (trial type presented in the previous trial) influences on motion perception. That is, besides the baseline condition with 100% consistent motion trials, one mixed condition was designed with 50% consistent motion and 50% inconsistent motion trials. Yet, for this mixed condition, the succession of trials was presented only quasirandomly in such a way that one half of the consistent motion trials was preceded by consistent motion trials, whereas the other half was preceded by inconsistent motion trials (the same was true for inconsistent motion trials). Therefore, the trial type in trial N-1 was added as an additional factor for the mixed condition, allowing for the systematic analyses of a potential influence of local trial type consistency. Both the baseline condition as well as the mixed condition were conducted twice. Once with the classical timing of 250-ms stimulus duration and interstimulus interval (e.g., Hubbard, 1990; Merz et al., 2019a, b), but also with a faster timing of 50 ms each, as this target timing is known to elicit stronger representational momentum effects (Hubbard & Bharucha, 1988; Merz, 2022).

## Method

### Participants

We selected forty participants for our prestudies to observe the expected medium-sized effect of trial type consistency on representational momentum. Additionally, we considered the possibility of a higher drop-out rate due to the use of an online, non-laboratory set-up. For a detailed explanation of our sample size rationale, please refer to Prestudy 1. Therefore, once again, a sample size of *N* = 40 was chosen. Six participants were excluded due to a high dropout of trials, presumably indicating a lack of engagement in the task (for more information, see the data-preparation section). The final sample (25 female, one diverse, eight male; three left-handed; mean age: 22.18 years, range: 18–38 years) consisted of 34 participants, all of whom were students at the University of Trier and participated in exchange for partial course credit. Six participants also participated in other experiments (five in Experiment 2, one in Experiment 4).^4^

### Design

Participants were tested in a two-factorial design with the within-participants factors of 2 (*experimental condition:* baseline condition—100% consistent motion vs. mixed condition—50% consistent motion) × 2 (*stimulus timing:* 250 ms vs. 50 ms), focusing on the hypothesis-relevant consistent motion trials. Additionally, for the mixed condition, a third within-participants factor of preceding trial type (consistent vs. inconsistent motion trial) was added. The participants were asked to estimate the final location of the visual target stimulus, and shift scores (difference between actual and estimated final location) were used as the dependent variable. Hereby, positive shift scores indicate a location estimation further along the motion trajectory (in other words, the representational momentum phenomenon), whereas negative shifts scores indicated a location estimation against the motion trajectory compared with the actual to-be-estimated location.

### Apparatus and stimuli

The participants used a computer or laptop of their choosing, no tablet, touchscreen or smartphones were allowed. If an operating system for a mobile device was detected, the experiment would not start. The experiment was programmed with PsychoPy and its built-in online translation PsychoJS (Peirce et al., 2019), data collection was via pavlovia.org. The final experimental set-ups were different for the participants (in the following, the number of participants is given in parentheses), yet, as this (as well as all of the subsequent) experiments were designed fully within-participants, these differences cannot explain any of the main finding reported in the manuscript. Participants used the touchpad of a laptop (19) or an external computer mouse (15) according to self-report (this was not detected by the experimental software, but participants were asked to indicate which set-up they have). As operating system, the Apple Mac OS (8), Linux (1) as well as Microsoft Windows (25), and as browser, Google Chrome (20), Mozilla Firefox (8) and Safari (6) were detected. All screens used a 60-Hz refresh rate, yet, resolutions different markedly between participants’ devices: 1,920 × 1,080 (5), 1,600 × 900 (1), 1,536 × 864 (8), 1,440 × 900 (7), 1,366 × 768 (6), 1,280 × 800 (1), 1,280 × 720 (5), 1,128 × 752 (1). The actual size of the screen (in inch or cm) was not assessed. Please note that representational momentum has been reliably shown to elicit comparable result in laboratory as well as online settings (Merz, 2022). The shape and size of the target was a 15 × 15 pixels white (RGB value: 255, 255, 255) circle on a grey background (RGB value: 127, 127, 127). A 15 × 15 pixels white (RGB value: 255, 255, 255) square was used to start the trial. As the experiments were conducted online, that is, with participants using their own laptop, and no control of the experimental situation, no chin rest was used, and no further instructions about seating position were given, as we would not have been able to control the implementation of these instructions.

### Procedure

At the beginning of each trial, participants were prompted to click on the square displayed at the centre of the screen in order to initiate the trial. The mouse cursor was presented in the form of the standard computer pointer. After the click was detected, the mouse cursor disappeared and a 600-ms blank interval was presented before the target was presented for the first time. After the target disappeared for the fifth and final time, a 500-ms blank interval was presented before the mouse cursor, displayed as a crosshair, appeared once again. The participant moved the crosshair to the center of perceived final location of the visual target stimulus and indicated this location by pressing the left mouse button (or touchpad), which ended the trial. Participants had to respond within 3,000 ms, otherwise the trial was terminated and a new trial began. Please note that the mouse cursor was only presented when an action was required (either clicking on the square to start a trial or when responding to the final location of the target stimulus), but was not displayed during the target presentation, as is typical for studies of representational momentum in order to prevent the mouse cursor from being used as a visual marker/landmark.

For the consistent motion trials, five successive presentations of the target stimulus (inducing stimuli) in the left-to-right or right-to-left direction, were presented (for a visualization, see Fig. 1). The horizontal distance between the successive presentations was fixed at 75 pixels. The final location of the visual target stimulus, that is, the fifth location of the target, which had to be estimated, was restricted to an 80x60 pixels window centred on the center of the screen (see location E in Fig. 1A). The y-axis value of the target stimulus was constant throughout one trial. Subsequently, the location of the first (second, third and fourth) presentation of the target stimulus was 300 pixels (225, 150, and 75 pixels) to the left (left-to-right motion direction) or right (right-to-left motion direction) of the final location for consistent motion trials.

For the inconsistent motion trials, the fifth (final) target location was chosen identically as for the consistent motion trials (see location E in Fig. 1A). The first, second, third, and fourth location of the target were selected randomly from eight possible locations (300, 225, 150, and 75 pixels to the left or right of the target—see locations A, B, C, D, F, G, H, I in Fig. 1A), while preventing consistent motion trials from being presented. No location was used twice during one trial (as is custom for inconsistent trials, e.g., Freyd & Finke, 1984; Merz et al., 2022). This gave rise to 1678 different inconsistent motion trials, from which the actual trial was selected at random.

Two experimental blocks were realized, one with only consistent motion trials (baseline condition), one with both trial types mixed (mixed condition—50% of each trial type). Both experimental blocks were realized twice, once for the same stimulus timing features as in classical representational momentum studies (ISI and stimulus duration of 250 ms), once for a fast stimulus timing (ISI and stimulus duration of 50 ms). Additionally, for the mixed block, 32 different quasirandom trial sequences were built, all of which had the same number of inconsistent motion and consistent motion trials preceding consistent motion trials. This allowed for the analysis of any influence of local trial consistency on motion perception. In all experimental blocks, the first trial, as well as the middle trial for the mixed block was designed to be excluded from analyses to have comparable number of trials for each to-be-analyzed condition. The actual trial sequence was selected (from the 32 quasirandom trial sequences) at random for each participant.

All instructions were provided in writing via the experimental software. Participants were instructed to indicate the center of the final/fifth location of the target. The participants worked through eight practice trials (randomly selected from all possible trials from the respective first block), before then completing 4 experimental blocks with overall 198 trials (66 trial for the 50% mixed block condition, although the first and 34^th^ trial were not analyzed; 33 trials for the 100% consistent motion block condition, although the first trial was not analyzed, both blocks were conducted twice, once with the slow, once with the fast stimulus timing condition; see the previous paragraph).

### Data preparation

If no response was detected within 3,000 ms of crosshair onset, then the participant was deemed to have failed to respond, and the trial was excluded from the data analysis. For each participant, trials in which the mouse cursor was not moved by the participant were removed. In these trials, the initial location (when the crosshair was presented after target presentation) and the final location of the mouse cursor (the location indicated by the participant with the help of the crosshair) was identical. It might have been the rare case that the estimation without any cursor movement was a conscious decision as the final location was perceived at the location at which the cursor appeared.^5^ However, it is far more likely that it was an accidental, erroneous mouse click or that it indicated a lack of engagement in the task. Participants needed to respond in order to get to the new trial, so just clicking the mouse without any movement constituted the fastest route to finishing the task. Due to these criteria, 7.66% of trials were excluded from data analysis. Additionally, the number of trials still included per participant was analyzed. Six participants were outliers considering the 1.5 interquartile range below the first quartile (Tukey, 1977). Therefore, they were excluded from data analysis. In a next step, shift scores were calculated. Shift scores indicate the difference between the actual and the estimated final location of the visual target stimulus along the horizontal x-axis (as the stimulus always moved horizontally; also known as M-displacement, displacement along the axis of motion, in the literature, e.g., Hubbard & Bharucha, 1988). A positive value indicates an overestimation in the direction of motion, whereas a negative value indicates an estimation against the direction of motion.

## Results

**Fig. 2:**
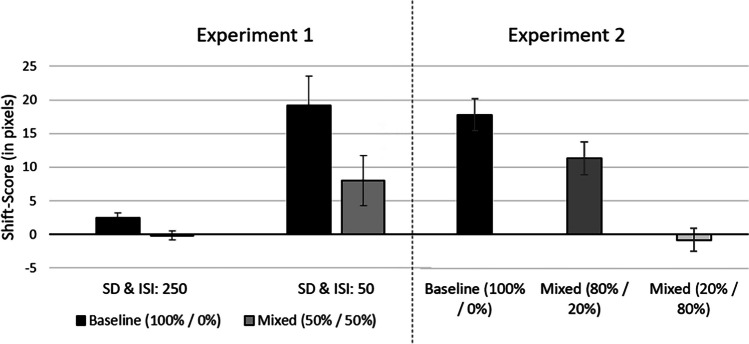
Results of Experiment 1 (left side) and Experiment 2 (right side)*.* For Experiment 1, two different experimental contexts (baseline condition and mixed condition) and two different timing conditions (slow condition with 250 ms, and a fast condition with 50 ms interstimulus interval and stimulus duration) were conducted. For Experiment 2, three different experimental context conditions (baseline condition, as well as two mixed conditions) were conducted. Hereby, one intermixed conditions consisted of 80% implied motion, and 20% inconsistent trials, or vice versa. In parentheses: First number indicates the percentage of implied motion trials within this experimental context, the second number indicates the percentage of inconsistent motion trials within this context. Errors bars represent standard errors of the mean. SD = stimulus duration; ISI: interstimulus interval

To analyze the data, a 2 (experimental condition: baseline condition vs. mixed condition) × 2 (stimulus timing: fast vs. slow) repeated-measures ANOVA was conducted. As expected and as can be seen in Fig. 2, a faster stimulus timing led to an increase of the forward shifts, and the indirect effect of trial type consistency was observed for both timing conditions, although it was stronger in the faster timing condition. That is, the main effect of stimulus timing, *F*(1, 33) = 13.42, *p* < .001, ɳ_p_^2^ = 0.29, and experimental condition, *F*(1, 33) = 16.09, *p* < .001, ɳ_p_^2^ = 0.33, as well as their interaction, *F*(1, 33) = 4.82, *p* = .035, ɳ_p_^2^ = 0.127, were significant. A forward shift for the slower timing condition for the baseline condition was observed (2.46 pixels), *t*(33) = 2.17, *p* = .037, *d* = 0.372. No effect was observed for the mixed condition for the slow timing (−0.15 pixels), *t*(33) = −0.23, *p* = .822, *d* = −0.039. For the faster timing condition, a significant forward shift was observed for both, the baseline block (19.20 pixels), *t*(33) = 4.37, *p* < .001, *d* = 0.750, as well as the mixed block (8.00 pixels), *t*(33) = 3.53, *p* = .001, *d* = 0.605.

In a next step, the focus was on a possible influence of local trial type consistency. That is, for the mixed block, a 2 (preceding trial *N* − 1: consistent motion vs. inconsistent motion) × 2 (stimulus timing: fast vs. slow) repeated-measures ANOVA was conducted. There was a main effect of stimulus timing, *F*(1, 33) = 5.40, *p* = .027, ɳ_p_^2^ = 0.14, but critically, no main effect of preceding trial, *F*(1, 33) = 1.43, *p* = .241, ɳ_p_^2^ = 0.04, nor any interaction, *F*(1, 33) = 0.23, *p* = .910, ɳ_p_^2^ < 0.01 (the respective descriptive statistics for the four factor combinations: consistent & fast: 8.40 pix; inconsistent & fast: 7.60 pix; consistent & slow: 0.33 pix; inconsistent & slow: −0.63 pix). To quantify the absence of an effect as indicated by the frequentist analysis, the same Bayesian ANOVA indicated comparable results, that is, the model with only the main effect of stimulus timing provided the best-fit, BF_M_ = 16.12, and was at least five times more likely than any model including the preceding trial *N* − 1, BF_01_ > 5.15.

## Discussion

Experiment 1 indicated the expected indirect influence of trial type consistency. In the baseline block with only consistent motion trials presented, the representational momentum phenomenon was observed, whereas for the mixed condition, the representational momentum effect was diminished (fast condition), or even eliminated (slow condition). Further, Experiment 1 allowed for a systematic analysis of a potential influence of preceding trial in the mixed block condition. Interestingly, the analyses indicated no influence of the preceding trial. That is, in the mixed condition, motion trials were perceived similarly, independent from whether the previous trial was a consistent or inconsistent motion trial.

## Experiment 2

Experiment 1 indicated that the local trial type consistency did not have an influence on perceived location for consistently moving stimuli. As such, it is likely that the global trial type consistency (the overall likelihood of the inconsistent trials within one experimental block) is the driving factor for the indirect influence of trial type consistency observed in the previous experiment. Therefore, Experiment 2 was designed specifically to test this hypothesis. Three different experimental conditions were realized. Once again, the baseline condition, with only consistent motion trials, and two mixed conditions were used. The first mixed condition consisted of 80% consistent motion trials and 20% inconsistent trials. For the second mixed condition, this mapping was reversed, that is, 20% consistent motion and 80% inconsistent motion trials were presented. The stimulus timing, as well as all other features of the task, were identical to the fast stimulus timing condition in Experiment 1 (50-ms ISI and stimulus duration), allowing for a better comparison between the experiments.

## Method

### Participants

Sample size calculation was identical to the previous experiment, yet, to allow for balancing of all block sequences (six different sequences) across participants, a sample-size of *N* = 42 was chosen. Seven participants were excluded due to a high dropout of trials, presumably indicating a lack of engagement with the task, as well as one participant for not following the rules of the task (for more information, see the data-preparation section). The final sample (30 female, zero diverse, four male; four left-handed, one ambidextrous; mean age: 22.06 years, range: 18–38 years) consisted of 34 participants, all of whom were students from the University of Trier and participated in exchange for partial course credit. Five participants had also taken part in Experiment 1.

### Design, apparatus and stimuli, procedure, and data preparation

The design, apparatus and stimuli, procedure, and data preparation were identical to fast (50-ms ISI and stimulus duration) condition in Experiment 1 with the following exceptions. Participants used the touchpad of a laptop (26) or an external computer mouse (9) according to self-report. As operating system, the Apple Mac OS (12), as well as Microsoft Windows (23), and as browser, Google Chrome (16), Mozilla Firefox (9) and Safari (10) were detected. All screens used a 60 Hz refresh rate, yet resolutions different markedly between participants: 1,920 × 1080 (2), 1,600 × 900 (1), 1,536 × 864 (8), 1,440 × 900 (10), 1,368 × 912 (1), 1,366 × 768 (5), 1,280 × 800 (3), 1, 280 × 720 (5).

The baseline condition (100% consistent motion trials) was exactly identical to Experiment 1. Both experimental blocks for the slow stimulus timing condition as well as the mixed condition for the fast stimulus timing condition were dropped. To analyze the global impact of the inconsistent trials, two mixed conditions were added. In one mixed condition (80%/20%), 80% consistent motion trials were mixed with 20% inconsistent motion trials. In the other mixed condition (20%/80%), 20% consistent motion trials were mixed with 80% inconsistent motion trials. For the mixed conditions, all possible trial types were selected at random.

The design of the experiment was adopted following these changes, and the participants were tested in a one-factorial design with the within-participants factor of condition (baseline vs. mixed [80%/20%] vs. mixed [20%/80%]). Trial numbers per condition were adopted to fulfil the abovementioned criteria regarding trial type proportions, participants responded to 24 consistent motion trials in each experimental block. Therefore, for the baseline block, 24 trials were included. For the mixed block (80%/20%), 30 trials (24 consistent motion trials, eight inconsistent motion trials) were conducted. For the second mixed block (20%/80%), 120 trials (24 consistent motion, 96 inconsistent motion trials) were included. Six versions were programmed to balance all possible sequences for the three experimental blocks, though the actual sequence was selected at random. Including six practice trials, participants responded to 180 trials overall (6 practice + 24 baseline + 30 mixed [80%/20%] + 120 [20%/80%]). The same data exclusion criteria as in Experiment 1 resulted in 6.26% of trial being excluded, and due to this, seven participants were excluded altogether. Additionally, for one participant, the estimated final location was within the possible final target range of the 80 × 60-pixel window centered on the middle of the screen in only 12 of all 180 trials. Additionally, this participant clicked closer to the onset location than to the offset location on average, indicating a lack of attending to the rules of the task. Therefore, this participant was also excluded.

## Results

The one-factorial ANOVA with the main effect of experimental condition (baseline condition vs. mixed condition 80%/20% vs. mixed condition 20%/80 %) revealed a significant main effect, *F*(2, 66) = 36.40, *p* < .001, ɳ_p_^2^ = .53. Directly comparing the three conditions, motion trials in the baseline condition revealed the strongest forward shifts (20.47 pixels), *t*(33) = 6.87, *p* < .001, *d* = 1.18, whereas no forward shift was observed 20%/80% mixed condition (−0.03 pixels), *t*(33) = −0.02, *p* = .990, *d* = −0.002. As Fig. 1 indicates, the forward shift diminishes with an increasing proportion of inconsistent motion trials mixed.

## Discussion

In Experiment 2, the proportion of inconsistent motion trials was systematically manipulated across three experimental blocks, that is, the proportion of consistent motion and inconsistent motion trials was manipulated within a given experimental block. The results clearly demonstrate that with an increasing proportion of inconsistent motion trials within one experimental block, the consistent motion trials are perceived very differently. That is, a large forward shift with a strong effect size (*d* = 1.18) in the baseline condition, can be completely eliminated when a large proportion of inconsistent motion trials are mixed. This is a remarkable change in the perception of physically identical trials resulting from the variation in the proportion of different trial types.

## Exploring the mechanism underlying the indirect influence of trial type consistency

The results of the first two experiments allowed us to describe the observed indirect effects of trial type consistency in more detail. That is, Experiment 1 ruled out a local, short-term influence of inconsistent trials on the perception of the classical consistent motion sequence. This indicates that the perceptual system tunes to the overall statistics of the experimental block, rather than using short-term adaptations of expectations. In line with this observation, the overall proportion manipulation of consistent and inconsistent motion trials had a strong effect on the perceived location of consistent motion trials. The questions arises about the mechanism underlying these observed results.

On the one hand, these results may be explained by the formulation of expectations, based on the target properties experienced within one experimental block. In other words, with the inclusion of inconsistent motion trials, the overall expectation regarding typical target speed or typical directional movement might have changed with the different proportions of inconsistent motion trials introduced within one experimental block. Therefore, the underlying driving factor would actually be changes of expectations introduced by the trial statistics introduced by the inconsistent trials. Subsequently, the overall proportion of consistent and inconsistent motion trials should not be the driving factor behind the observed results in Experiments 1 and 2, but rather the trial statistics introduced by the inconsistent motion trials. Alternatively, it might be argued that with the decrease in the overall proportion of consistent trials, the reliance on the mechanism leading to the representational momentum phenomenon is simply attenuated (e.g., the motion vector in motion direction, Hubbard, 1995; or a change from active tracking of the target with only consistent motion trials to a passive/not at all tracking of the target with inconsistent trial intermixed, Thornton et al., 2002,6). In other words, if consistent motion is less likely to occur, the perceptual system does not rely as heavily on the typical mechanism underlying motion perception as it would in a block when all trials are classical consistent motion trials. This explanation would point toward the overall proportion of consistent and inconsistent trials influencing motion perception, but not the trial statistics introduced by the inconsistent motion trials. Therefore, Experiment 3 and 4 were designed to differentiate between these two suggestions, by presenting two mixed context with 50% consistent and inconsistent motion trials, yet, in which the stimulus characteristics for the inconsistent motion trials were systematically manipulated. Following the expectation hypothesis, these experimental contexts should result in different shift scores, whereas the attenuation idea would expect no change regarding the shift scores.

## Experiment 3

**Fig. 3:**
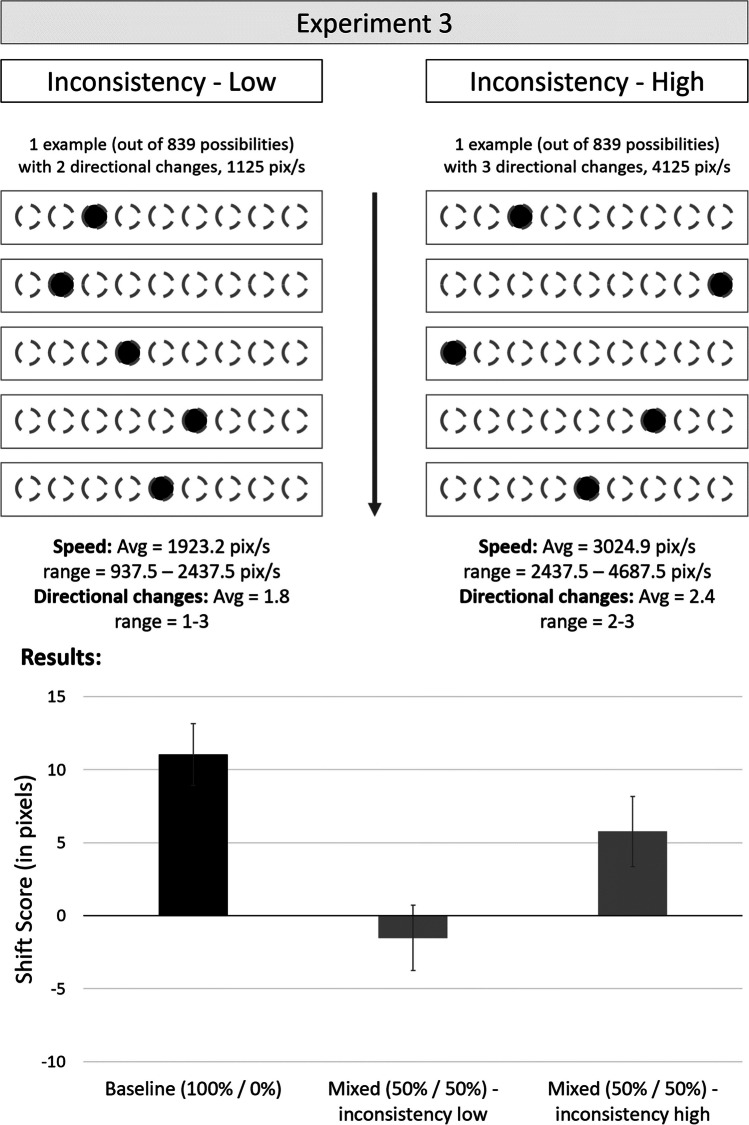
Experimental logic (top) and results (bottom) for Experiment 3. For Experiment 3, two mixed conditions were designed, with differing amount of inconsistency regarding directional changes and target speed. One example for the low (left) and high (right) inconsistency trial types are shown, with ranges and averages presented below the trial examples. On the bottom, shift score results for the three experimental conditions (baseline vs. mixed—inconsistency low vs. mixed—inconsistency high) are presented. Errors bars represent standard errors of the mean

Experiment 3 was designed to differentiate between the two hypotheses—the attenuation hypothesis on the one hand and the expectation hypothesis on the other. Therefore, two mixed conditions with a comparable proportion of consistent motion and inconsistent motion trials were designed, both with 50% consistent motion and 50% inconsistent motion trial types. Importantly, the inconsistent trials for the two mixed conditions were selected from two different distributions. That is, for one mixed block, only inconsistent motion trials indicating very fast speeds, that is, on average large jumps between successive target presentations, were selected (inconsistency—high; for a visualization, see Fig. 3). For the other mixed condition, only inconsistent motion trials, indicating rather low speeds, that is, on average, short jumps between successive target presentations, were selected (inconsistency—low; see Fig. 3). Due to our restriction of the nine possible target locations (see Fig. 1A), faster target speeds should correlate with higher amounts of directional changes, which is the subject of Experiment 4. Therefore, if both mixed conditions resulted in different perceptual biases, this would support the notion of expectations being the driving force behind the observed results, as the two experimental blocks were designed to form different expectations (due to the manipulation of high or low inconsistency). In contrast, if both mixed conditions resulted in similar data pattern, this would support the attenuation hypothesis (as the overall proportion of consistent and inconsistent trial types were identical for both experimental blocks with 50% each). As in the previous experiments, the baseline condition was included.

## Method

The sample size calculation was identical to Experiment 2. Five participants were excluded due to a high dropout of trials, presumably indicating a lack of engagement in the task, as well as two participants for not following the rules of the task (for more information, see the data-preparation section). The final sample (24 female, zero diverse, 11 male; three left-handed, zero ambidextrous; mean age: 22.46 years, range: 18–35 years) consisted of 35 participants, all of whom were students from the University of Trier and participated in exchange for partial course credit. Five participants also participated in Experiment 4.

### Design, apparatus and stimuli, procedure, and data preparation

The design, apparatus and stimuli, procedure, and data preparation were identical to Experiment 1, with the following exceptions. Participants used the laptop touchpad (19) or an external computer mouse (16) according to self-report. In terms of the operating system, the Linux (1), Apple Mac OS (11), as well as Microsoft Windows (23), and as browser, Google Chrome (16), Mozilla Firefox (12), and Safari (7) were detected. All except one screen used an approximate 60 Hz refresh rate, yet, resolutions different markedly between participants: 2,240 × 1,260 (1), 1,920 × 1,080 (3), 1,680 × 1,050 (2), 1,536 × 864 (7), 1,440 × 900 (8), 1,366 × 768 (5), 1,280 × 800 (2), 1,280 × 720 (6), 1,128 × 752 (1).

Only the baseline block condition with fast stimulus timing (50 ms stimulus duration and ISI) from Experiment 1 was kept. Furthermore, the fast stimulus timing condition for the mixed condition from Experiment 1 was adapted slightly. That is, in the previous two experiments, the actual inconsistent motion sequence was selected out of a possible 1,678 different inconsistent motion trials. For each of the consistent motion and inconsistent motion trials, the actual speed was calculated. That is, four distances (from the first to the second, from the second to the third, from the third to the fourth, and from the fourth to the fifth and final location) were calculated and averaged. Then, given the stimulus timing of 50 ms ISI and stimulus duration, each actual speed was calculated. For the consistent motion sequence, this resulted in 750 pix/s (with a distance between successive presentations fixed at 75 pixels, the smallest possible distance). In contrast, for the inconsistent motion patterns, the extracted speeds differed markedly, resulting in speeds ranging between 937.5 pix/s and 4687.5 pix/s. Subsequently, all inconsistent motion trial possibilities were split in half, and for one mixed condition, only slower inconsistent trials were presented (ranging from 937.5 pix/s to 2437.5 pix/s; inconsistency low condition). For the other mixed condition, only faster inconsistent motion trials were presented (ranging from 2437.5 pix/s to 4687.5 pix/s; inconsistency high condition). Importantly, for both experimental blocks, the proportion of consistent motion and inconsistent motion trial was kept constant with 50% each.

Participants were tested in a one-factorial design (condition: baseline vs. mixed—inconsistency low vs. mixed—inconsistency high). As with the previous experiments, 24 consistent motion trials were presented per condition. Subsequently, the mixed conditions consisted of 48 trials overall, and with 8 practice trails, the overall trial count for the experiment was at 128 trials (8 practice trials + 24 baseline trials + 48 trials mixed - inconsistency low + 48 trials mixed - inconsistency high). The same data exclusion criteria resulted in 4.77% of trials being excluded, and due to this, five participants were excluded altogether. Additionally, two participants clicked closer to the onset location than to the offset location on average, indicating a lack of attending to the rules of the task, and so they were also excluded.

## Results

First the inconsistent trials for the two mixed conditions were checked to ensure that the experimental manipulations actually gave rise to experimental blocks with different characteristics regarding the inconsistent motion trials. As expected, for the low inconsistency condition, the average speed for the inconsistent trials was slower than in the high inconsistency condition (1,923.2 pix/s vs. 3,024.9 pix/s), *t*(34) = 51.61, *p* < .001, *d* = 8.72. Additionally, the number of direction changes in the low consistency condition was, on average, only 1.8 directional changes, whereas for the high consistency condition, the number of direction changes was, on average, 2.4 directional changes, *t*(34) = 23.11, *p* < .001, *d* = 3.91. That is, more directional changes are associated with higher overall speed because of the restrictions of possible target locations within our experimental design, which will be the subject of Experiment 4. In a next step, the one-factorial ANOVA with the main effect of experimental condition (baseline vs. low inconsistency vs. high inconsistency) revealed a significant main effect, *F*(2, 68) = 16.00, *p* < .001, ɳ_p_^2^ = .32. Yet, crucially, our interest was particularly on the two mixed conditions (for a visualization, see Fig. 3). Here, the consistent motion trials in the high inconsistency condition resulted in a forward shift (5.8 pix), *t*(34) = 2.58, *p* = .014, *d* = 0.44, but not in the low inconsistency condition (−1.5 pix), *t*(34) = −0.63, *p* = .535, *d* = −0.11. Directly comparing both conditions, this resulted in a significant difference, *t*(34) = 2.81, *p* = .008, *d* = 0.48. This result clearly indicates that the overall proportion of consistent and inconsistent trials was not the driving factor in the observed results, but that the amount of inconsistency introduced by the inconsistent trials was the relevant moderating factor.

## Discussion

The results of Experiment 3 support the notion that expectations regarding the actual, to-be-encountered target stimulation are used by our perceptual system. That is, we were able to observe a difference for the two mixed conditions based on differences of inconsistency, while keeping the overall proportion of consistent motion and inconsistent motion trials constant. This argues against a proportion-based account (such as formulations of an attenuation mechanism), and in favour of expectations-based explanations for motion perception instead.

## Experiment 4

**Fig. 4:**
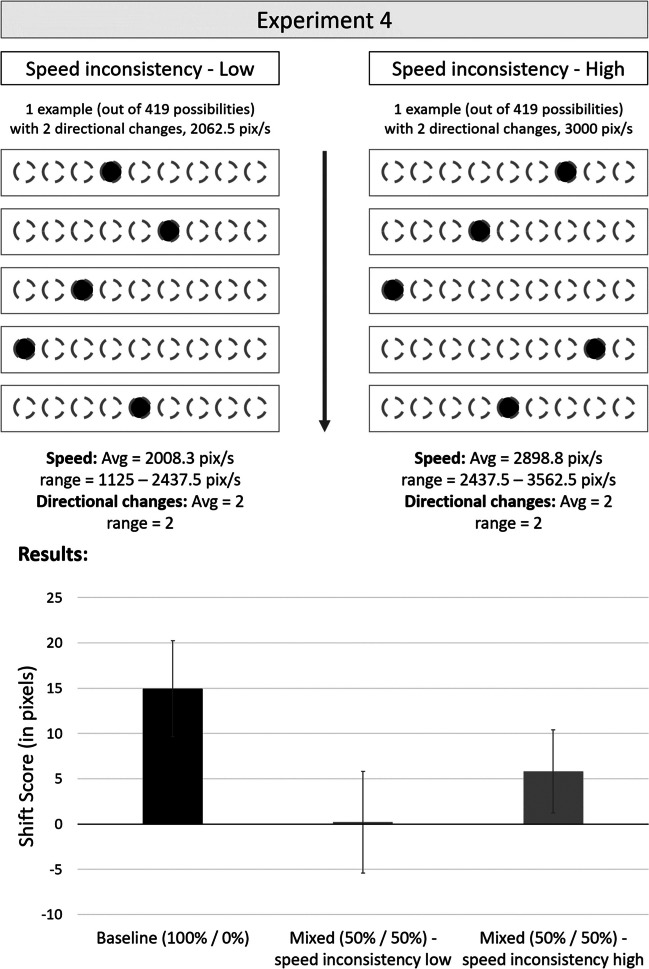
Experimental logic (top) and results (bottom) for Experiment 4. For Experiment 4, two mixed conditions were designed, with differing amount of inconsistency regarding target speed, while keeping directional changes constant at 2. One example for the low (left) and high (right) speed inconsistency trial types are shown, with ranges and averages presented below the trial examples. On the bottom, shift score results for the three experimental conditions (baseline vs. mixed—speed inconsistency low vs. mixed—speed inconsistency high) are presented. Errors bars represent standard errors of the mean

The question arises about the nature of these expectations formed by the perceptual system. More precisely, the inconsistent motion trials differ from the consistent motion trials in two key ways: consistency regarding motion direction and consistency regarding induced speed. In the previous experiment, the high inconsistency condition differentiated from the low consistency condition in the number of changes in motion direction as well as in induced speed (for a visualization, see Fig. 3). Therefore, in this final experiment, the experimental logic was kept similar to that of Experiment 3, except that for the two mixed conditions, they were not only equated regarding trial type proportion (50% consistent motion, 50% inconsistent motion trial types), but also regarding directional changes for the inconsistent motion trials (for a visualization, see Fig. 4). That is, all inconsistent motion trials always changed direction twice, while still indicating different speeds. Therefore, if a difference between the two mixed conditions is still observed, this result can only be due to the speed characteristics of the inconsistent motion trials.

## Method

The sample size calculation was identical to the previous Experiment 3. Due to an error in data collection, the data from one additional participant was collected. Three participants were excluded due to a high dropout of trials, presumably indicating a lack of engagement in the task (for more information, see the data-preparation section). The final sample (31 female, zero diverse, nine male; eight left-handed, zero ambidextrous; mean age: 22.05 years, range: 18–35 years) consisted of 40 participants, all of whom were students from the University of Trier and participated in exchange for partial course credit. Six participants also participated in other Experiments (five in Experiment 3, one in Experiment 1).

### Design, apparatus and stimuli, procedure, and data preparation

The design, apparatus and stimuli, procedure, and data preparation were identical to Experiment 3 with the following exceptions. Participants used the touchpad of a laptop (28) or an external computer mouse (12) based on self-report. As operating system, Linux (1), the Apple Mac OS (17), as well as Microsoft Windows (22), and as browser, Google Chrome (19), Mozilla Firefox (9), and Safari (12) were all detected. Most of the screens had an approximate 60-Hz refresh rate (33), some had 30 Hz (3), one had 72 Hz, one had 100 Hz, and two had 111 Hz. Screen resolutions differed between participants: 2,240 × 1,260 (1), 1,920 × 1,080 (3), 1,680 × 1,050 (1), 1,536 × 864 (8), 1,504 × 1,003 (2), 1,440 × 960 (1), 1,440 × 900 (14), 1,368 × 912 (3), 1,366 × 768 (2), 1,280 × 800 (1), 1,280 × 720 (4).

The experimental blocks were identical to Experiment 3, except that only inconsistent motion trials with exact two directional changes in one trial sequence were used. That is, 838 out of the original 1,678 inconsistent patterns were identified which indicated two directional changes during one trial (speed ranging from 1,125 pix/s to 3,562.5 pix/s). As for Experiment 3, these trials were ordered based on their indicated speed and split into two halves. The faster trials were then used for the high speed inconsistency condition, the slower trials were used for the low speed inconsistency condition. The same data exclusion criteria resulted in 2.97% of trials being excluded, and due to this, three participants were excluded altogether.

## Results

At first, as in Experiment 3, we checked that the inconsistent motion trials for the two mixed conditions actually resulted in different speed characteristics for the inconsistent motion trials. As expected, for the low speed inconsistency condition, the average speed in the inconsistent motion trials was slower than in the high speed inconsistency condition (2,008.3 vs. 2,898.8 pix/s), *t*(39) = 50.95, *p* < .001, *d* = 8.05. Subsequently, the one-factorial ANOVA with the main effect of experimental condition (baseline vs. low speed inconsistency vs. high speed inconsistency) revealed a significant main effect, *F*(2, 68) = 14.8, *p* < .001, ɳ_p_^2^ = .28. Yet, crucially, the interest was on the two mixed conditions (for a visualization, see Fig. 4). Even though the consistent motion trials in the high speed inconsistency condition revealed descriptively the same forward shift in terms of its magnitude (5.8 pix), this was not statistically significant, *t*(39) = 1.27, *p* = .212, *d* = 0.21, and no shift was observed in the low speed inconsistency condition (0.2 pix), *t*(39) = 0.04, *p* = .972, *d* = 0.01. Crucially, when directly compared, the consistent motion trials in the high speed inconsistency condition (5.8 pix) were significantly different from the consistent motion trials in the low speed inconsistency condition (0.2 pix), *t*(39) = 2.13, *p* = .039, *d* = 0.337.

## Discussion

The results of Experiment 4 help to resolve the question regarding the nature of the observed indirect influence of trial type consistency observed in the present manuscript. That is, the changes of induced speed introduced by the inconsistent trial types resulted in the perceived change of moving stimuli. That is, a difference between the two mixed conditions was observed in the data, yet, the two experimental blocks only differed in terms of the speed profile of the inconsistent trial types, but not regarding the directional changes induced by the inconsistent motion trials. This result indicates that expectations regarding stimulus speed are used to inform the final percept of dynamic, moving stimuli.

## General discussion

Across four experiments, the nature of expectations influencing the perception of moving stimuli was analyzed by exploring the perception of consistent motion trials in different experimental blocks. While in the first two experiments, the data indicated that the observed effect was an effect of global, not local, trial type consistency, the next two experiments helped to pinpoint the observed effect to be driven by the change of speed characteristics, induced by the inconsistent motion trials. This indicates that the perceptual system uses the speed characteristics of the current experimental block, likely by forming expectations regarding these circumstances to inform the final percept. This notion is in line with theories arguing for the importance of speed expectations for human motion perception (such as Merz et al., 2022).

The observed modulation of the perceived final location in the present study is likely to originate perceptually, in line with expectation-based theories such as the speed prior account (Merz et al., 2022, see also Merz et al., 2023). Previous evidence has shown that the frequency with which different responses are given block is likely to be equated across an experimental block (e.g., Erlebacher & Sekuler, 1971). Yet, it should be noted that the experimental set-up used here was designed in such a way that response changes within our different experimental blocks cannot provide an alternate explanation for the findings. The final stimulus location, which had to be estimated by the participants with the help of a computer mouse, was randomly distributed around the central location of the screen. Crucially, this was identical for consistent motion as well as inconsistent motion trials, making any moderating influence of target location, and subsequent response motion toward the perceived target location, very unlikely.

The results reported here provide a possible explanation for a puzzling finding concerning the phenomenon of tactile representation momentum (see, relatedly, Merz et al., 2023). That is, while initially no representational momentum was observed for tactile stimulation (e.g., Macauda et al., 2018; Whitsel et al., 1986), subsequent studies evidenced a tactile analogue of this phenomenon (e.g., Merz et al., 2019a, b), even though fairly similar stimulus speeds were used in the majority of these studies (about 6–7 cm/s along the forearm). Surprisingly, and in contrast to the present results, representational momentum is only observed in touch when consistent motion and inconsistent motion trials are mixed, but not when presented in isolation. These differences between sensory modalities might possibly be explained by arguing for different sensory acuities (higher spatial acuity in vision, especially for foveal vision, compared with touch, e.g., Gallace & Spence, 2014; Pick et al., 1969; Sheth & Shimojo, 2001; Wässle et al., 1990; Weinstein, 1968; resulting in differences of encoding of stimulus speed and subsequent updating of the speed expectation) and/or by pointing toward different typical speed statistics expected a priori for the two senses (e.g., tactile sensations are different/non-overlapping with visual sensations, therefore different a priori expectations might exist for the different sensory modalities).

While, in general, the present results are in line with the idea of speed expectations influencing the perception of motion stimuli, the complete data set as evidenced in this study cannot be accounted by any theory as currently proposed in the literature, including the speed prior account (Merz et al., 2022). That is, for Experiment 3 (and 4), the high (speed) inconsistent condition indicated a faster stimulus speed compared with the low (speed) inconsistent condition, therefore with the baseline (100% consistent motion trials) condition evidencing the strongest forward shifts, it would have been expected that the high (speed) inconsistent leading to the weakest/no forward shifts, and the low (speed) inconsistent trials to intermediate scores. Yet, the reverse pattern was observed. Although the speed prior account argues against a linear increase of the representational momentum phenomenon with increasing stimulus speed, but more so for a curvilinear/partially quadratic connection, the observed change of results would be difficult to be predicted a priori. Therefore, the results presented here also argue for a refinement of already existing, or else the development of entirely new, theoretical accounts.

The results reported here should be differentiated from previous findings showing influences of experimental context by other information that is presented simultaneously with the response relevant target stimulus. In these studies, other stimulus material (such as a second/another stimulus, spatial or directional cues or so) are presented simultaneously to investigate the direct effect of contextual information (for overview and discussion, see Hubbard, 2005, 2014). Yet, in the present study, we were interested in the indirect effect of trial type consistency, more precisely, the possible changes introduced by the manipulation of trial types statistics. The data that have been presented here indicate that the overall compilation of the experimental situation has a potential enormous effect on the subsequent perception of stimuli. “What influence does the inclusion of a specific trial type have?” is a crucial question for experimental researchers to ponder when designing experiments.

Our findings can be linked to theoretical ideas of curiosity exploration of our cognitive system, originally formulated by Berlyne (1949, 1960; for a recent study, see Frings et al., 2019), or the idea of surprise reductions (see Friston, 2009). That is, our perceptual/cognitive system is designed to explore variation in our surroundings to adapt its expectations within this experimental setting to allow for context optimal perception and action. That might be why the speed statistics are updated within the different experimental blocks that were presented in our study, but might very well be a general goal of our cognitive system to act optimally.

## Conclusions

The present study reports evidence across four experiments that the stimulus statistic of trials included in an experimental block plays a central role during the perception of dynamic stimuli. By analyzing only perceptually identical trails, we were able to show a crucial influence of stimulus statistics on motion perception. Furthermore, the results indicate a possible influence of speed expectation about actual, to be expected target motion on motion perception.

## Data Availability

Raw data, experimental files and important analyses scripts are publicly available (https://osf.io/q25mw/?view_only=38e3985735384bcc937eade8aaed9532). A demonstration of the trials used in the experiment can be accessed here: (https://run.pavlovia.org/S_Merz/consistency_demo/html). None of the experiments reported here was preregistered.

## Appendix 1: Exploratory analysis of inconsistent motion trials

In this section, the localization of the inconsistent motion trials are analyzed. In the main manuscript, we argue that introducing the inconsistent motion trials altered the stimulus statistics, particularly affecting the speed statistics within any experimental block. This change consequently influenced the speed expectations in that block. As such, the localization of the inconsistent motion trials should also be systematically influenced according to the predictions of speed-expectations-based accounts such as the speed prior account (Merz et al., 2022), which is subject for this exploratory analysis. Specifically, this account predicts a curvilinear or quadratic relation between speed and the shift score, characterized first by an increase with increasing speed, which later transitions to a backward shift at faster speeds. Given the considerably faster speed in the present manuscript, ranging between 937.5 and 4687.5 pix/s, a tendency towards a backward shift was expected, particularly for faster stimulus speeds.

The inconsistent motion trials are distinct from the consistent ones regarding both directional and speed consistency. The directional inconsistency, wherein both left-to-right and right-to-left movements were presented within a single trial, necessitate an explanation of how the dependent variable is computed. Typically, the representational momentum effect or forward shift is understood as the overestimation in motion direction. We maintain that the decisive direction is the final one, tracing from the penultimate to the last location, as it marks the motion direction just before cessation and localization. Consequently, in this exploratory analysis, shift scores indicate either a forward shift in the direction of the final target motion (positive values) or a backward shift against it (negative values). Similarly, the final target speed was considered as the determining factor, derived from the spatial separation between the penultimate and last target location relative to the stimulus timing. Given that the final speed could be 750 pix/s, 1,500 pix/s, 2,250 pix/s, or 3,000 pix/s, grouped, to increase power (as final target speed was not systematically balanced across the presented inconsistent motion trials), into a slow final target speed (750 pix/s and 1500 pix/s) and a fast final target speed group (2,250 pix/s and 3,000 pix/s).^7^

For Experiment 1, the inconsistent trials in the mixed blocks for the slow (250-ms ISI and stimulus duration) and fast (50-ms ISI and stimulus duration) stimulus timing condition were analyzed with a 2 (stimulus timing: 250 ms vs. 50 ms) × 2 (final target direction: left-to-right vs. right-to-left) × 2 (final target speed: slow vs. fast) ANOVA. Overall, a significant backward shift, that is, offset repulsion, is observed, *t*(33) = −2.82, *p* = .008, *d* = −0.48. None of the effects was significant, *F* < 3.32, *p* > .077, except for the main effect of final target speed, *F*(1, 33) = 7.78, *p* = .009, ɳ_p_^2^ = .19, with no effect for the slow final target speed (−1.15 pix), *t*(33) = −0.80, *p* = .428, yet, a strong backward shift for the fast final target speed conditions (−6.44 pix), *t*(33) = −3.50, *p* = .001, *d* = 0.60, in line with the predictions of the speed prior account. The same results were mirrored in Experiment 2, where, only the mixed block with 80 % inconsistent motion trials was analyzed, as in the other mixed block (with 20% motion trials), only six inconsistent trials were presented to each participant. In the 2 (final target direction: left-to-right vs. right-to-left) × 2 (final target speed: slow vs. fast) ANOVA, once again the main effect of final target speed was significant, *F*(1, 33) = 7.30, *p* = .011, ɳ_p_^2^ = .18, once again showing more negative shifts for the fast condition (−10.54 pix) then in the slow condition (−2.38 pix). Additionally, the main effect of final target direction was significant, *F*(1, 33) = 5.29, *p* = .028, ɳ_p_^2^ = .14, with more negative values for the right-to-left direction (−11.51 pix) then the left-to-right direction (−1.41).

For Experiments 3 and 4, the factor of experimental condition (low [speed] inconsistency vs. high [speed] inconsistency) was added as an additional within-participants factor to the 2 (final target direction: left-to-right vs. right-to-left) × 2 (final target speed: slow vs. fast) ANOVA. For Experiment 3, none of the main effects nor the interaction was significant, *F* < 1.48, *p* > .232, although the descriptive shifts scores for the slow (−3.93 pix) and fast (−7.79 pix) were once again in the same direction as the results observed in Experiments 1 and 2. For Experiment 4, the same 2 × 2 × 2 ANOVA once again revealed the significant main effect of final target speed, *F*(1, 39) = 7.31, *p* = .010, ɳ_p_^2^ = .16, showing more negative values for the fast final target speed (-12.65 pix) than for the slow final target speed (−1.95 pix). Additionally, this main effect was modulated by the experimental condition, *F*(1, 39) = 7.27, *p* = .010, ɳ_p_^2^ = .16. In the high inconsistency condition, this difference between fast (−7.19 pix) and slow (−4.73 pix) was less pronounced than in the low inconsistency condition (fast: −18.12 pix; slow: 0.84 pix).

In general, the analysis of the inconsistent motion trials across the four experiments reveals two central data patterns: First, the overall tendency to indicate negative shift scores, that is, an offset repulsion effect. This might be surprising, especially for readers familiar with the original study by Freyd and Finke (1984), in which the authors did not observe any effect for inconsistent motion trials (Experiment 2 of that study; although, a closer inspection of the RTs in this study also indicated a descriptive, but non-significant tendency toward a backward shift). Yet, from the perspective of the speed prior account, these results are expected, given that the representational momentum effect will start to decrease after some initial increase with faster stimulus timing. Please also note that compared with Freyd and Finke’s (1984) study, we used much faster stimulus timing (50 ms ISI as well as stimulus duration) than was used in the original study (250 ms ISI and stimulus duration). Second, a stronger offset repulsion effect was observed for the faster final target speed conditions across all four experiments (this effect was nonsignificant, although descriptively similar, in Experiment 3) compared with the slow final target speed condition. This is in line with formulations arguing for the importance of speed to influence target localization and perception, such as the speed prior account (Merz et al., 2022).

## Footnotes

1 Kerzel (2002) proposed that representational momentum was only observed with targets that were predictable, that is, in those tasks in which either stimulus direction or the final location of the object was predictable/fixed in an experimental context. Note that this claim is in doubt, given that representational momentum has also been observed when stimulus direction and the actual vanishing point are not fixed within a given experimental context; see Hubbard (2005), for discussion; see also the present study.

2 For a detailed discussion and the analysis of inconsistent motion trials within our experiments, see the Appendix 1.

3 The usage of an online version of the representational momentum task likely introduces different experimental setups, and subsequent different stimuli presentations regarding stimulus size in visual angle. Yet, our experiments were explicitly designed to have all manipulations and subsequent comparisons within-participants in order to prevent any meaningful influence of experimental setup on our results. Please also note that horizontal, implied motion sequences to investigate representational momentum have been previously shown to elicit very comparable data patterns in an online and in a laboratory setting (Merz, 2022).

4 In this study, a few participants took part in more than one experiment. Yet, follow-up analysis for all four experiments excluding any participant who participated in more than one experiment did not significantly change the results reported in the main text.

5 The final location was restricted to an 80x60 pixel window centred on the center of the screen. Therefore, 4,800 possible final locations existed, but participants only responded to 288 experimental trials, resulting in a 6% chance for each participant that one such trial occurred during their experiment.

6 We thank Ian Thornton for pointing us toward this potential alternative explanation.
